# Geriatric Nutritional Risk Index and mortality in individuals with prediabetes and diabetes: a longitudinal cohort study

**DOI:** 10.3389/fnut.2025.1625281

**Published:** 2025-07-15

**Authors:** Luyao Qiao, Te Li, Jiaxing Peng, Qing Xie, Mengqian Wu, Yanping Li, Zhenyu Tang

**Affiliations:** ^1^Department of Neurology, The Second Affiliated Hospital, Jiangxi Medical College, Nanchang University, Nanchang, China; ^2^Department of Nephrology, Tongji Hospital, School of Medicine, Tongji University, Shanghai, China

**Keywords:** nutritional status, Geriatric Nutritional Risk Index, diabetes, prediabetes, all-cause mortality, cardiovascular mortality

## Abstract

**Background:**

This study examines the relationship between Geriatric Nutrition Risk Index (GNRI) and all-cause and cardiovascular mortality in individuals with prediabetes and diabetes, aiming to guide clinical nutrition management and extend life expectancy.

**Methods:**

We analyzed a weighted sample of 7,640 individuals with prediabetes and diabetes from the NHANES 2005–2018 and the NCI database. Nutritional status was assessed using the GNRI. Survival outcomes, including all-cause and cardiovascular mortality, were analyzed using Cox proportional hazards regression models and Kaplan–Meier survival curves. Subgroup analyses and restricted cubic spline (RCS) regressions were further conducted to evaluate the robustness and potential nonlinear relationships between GNRI and mortality outcomes.

**Results:**

Over a median follow-up of 8.00 years, 1,210 participants died, including 319 from cardiovascular diseases. Kaplan–Meier curves revealed significantly lower survival rates for both mortalities in participants with low GNRI. Fully adjusted COX regression models revealed a 2.50-fold (95% CI: 2.14–2.92, *p* < 0.001) increased risk of all-cause mortality and a 2.78-fold (95% CI: 2.04–3.77, *p* < 0.001) increased risk of cardiovascular mortality in the low GNRI group. These associations remained robust across subgroup analyses. RCS analyses presented nonlinear associations between GNRI and both mortalities (both *p*-non-linear <0.05, *p*-overall <0.05).

**Conclusion:**

GNRI demonstrated a significant, negative, and nonlinear association with all-cause and cardiovascular mortality in US individuals with prediabetes and diabetes, highlighting its utility in improving survival outcomes through nutritional assessment.

## 1 Introduction

The global prevalence of diabetes and prediabetes has reached alarming levels, posing a major public health crisis. Factors including aging populations, urbanization, reduced physical activity, and rising obesity rates contribute to this upward trend (1). The most recent IDF Diabetes Atlas estimates that 537 million people worldwide are living with diabetes, with projections reaching 700 million by 2045 (2). Additionally, prediabetes, characterized by impaired glucose tolerance and an intermediate stage between normal glucose regulation and diabetes, affects an even larger portion of the population and carriers a significant risk of progression to metabolic syndrome (MetS) and diabetes. Same as MetS, diabetes is a leading cause of mortality, with individuals facing a 2–3 times higher risk of all-cause mortality (3, 4), particularly from cardiovascular diseases (4–6). It underscores the importance of early intervention and management for this population.

Malnutrition represents a critical modifiable factor in the progression and clinical outcomes of prediabetes and prediabetes. Emerging evidence highlights its complicated impact, exacerbating disease severity via metabolic dysregulation while escalating microvascular/macrovascular complication risks (7, 8). This imbalance perpetuates glycemic instability and accelerates end-organ damage, forming a pathogenic cycle in diabetic patients. Recent studies have linked poor nutritional status in diabetic patients to higher morbidity and mortality through mechanisms such as neurological inflammation, oxidative stress, endothelial dysfunction, and intestinal microbiota (9–11). While obesity is a primary driver of MetS and a significant contributor to the pathogenesis of diabetes and cardiovascular diseases (12), these findings underscore the need for a holistic approach to dietary research, focusing on systemic nutritional studies rather than isolated dietary components to better evaluate dietary patterns’ synergistic metabolic health impacts (13).

The Geriatric Nutritional Risk Index (GNRI), introduced by Bouillanne et al. (14), combines serum albumin levels with actual and ideal body weight to assess nutritional status. GNRI has been shown to predict outcomes in various diseases, including diabetes (15), osteoporosis (16), prostate cancer (17), and chronic obstructive pulmonary disease (18), and is considered more reliable and less influenced by subjective factors than other tools like the Nutritional Risk Score (NRS-2002) and the Malnutrition Universal Screening Tool (MUST) (14, 17, 19, 20). Despite its established relevance in other conditions, there is limited evidence regarding the role of GNRI in the risk of all-cause and cardiovascular mortality in individuals with diabetes or prediabetes. Additionally, research on the nutritional status of participants with prediabetes is scarce. Therefore, this study aims to investigate the link between GNRI and all-cause and cardiovascular disease mortality among individuals with diabetes and prediabetes, filling a critical gap in the literature. Our findings will provide valuable insights into optimizing clinical nutrition management and improving prognosis in this high-risk population.

## 2 Methods

### 2.1 Participants

The NHANES, authorized by the Centers for Disease Control and Prevention, has aimed to provide a comprehensive and nationally representative sample to evaluate the health and nutrition of individuals in the United States in two-year cycles since 1999. The database contains information on demographics, diet, examination, laboratory tests, and questionnaires. The project adhered to the principles of the Declaration of Helsinki. Prior to taking part in the survey, all individuals provided their consent.

**Figure 1:**
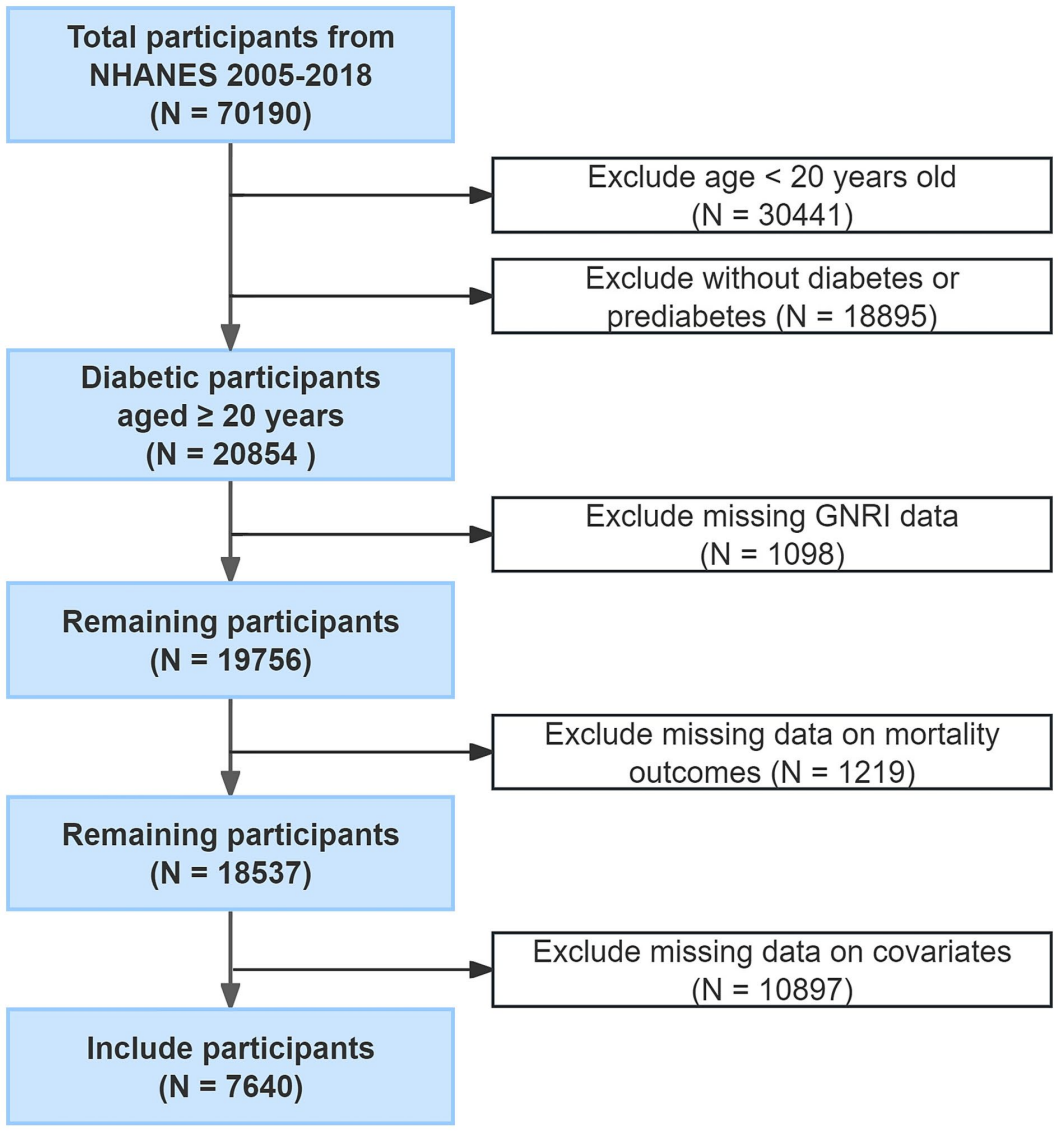
Flowchart for the selection of participants.

Initially, a total of 70,190 individuals based on seven survey cycles of the NHANES database from 2005 to 2018 participated. We enrolled participants with diabetes or prediabetes aged ≥20 years (*n* = 20,854). Individuals with missing data on GNRI (*n* = 1,098), mortality details (*n* = 1,219), and several covariates, including gender, age, education level, race, marital status, PIR, BMI, smoking status, and alcohol consumption (*n* = 10,897) were excluded from the analysis. Ultimately, 7,640 individuals with diabetes or prediabetes were enrolled in the final analysis (Figure 1). According to the American Diabetes Association (ADA) diagnosis and classification standards (21), participants with diabetes were defined as meeting any of the following criteria: fasting plasma glucose (FPG) ≥126 mg/dL, 2-h plasma glucose (2-h PG) ≥200 mg/dL, or glycated hemoglobin (HbA1c) ≥6.5%. Participants having self-reported doctor diagnosed diabetes (as indicated by the question “Doctor told you have diabetes” in the NHANES diabetes questionnaire) were also defined as having diabetes (22, 23). Patients with prediabetes were defined as meeting any of the following criteria: FPG between 100 and 125 mg/dL, 2-h PG between 140 and 199 mg/dL, or HbA1c between 5.7 and 6.5% (21).

### 2.2 Assessment of GNRI

We chose the GNRI to assess the nutritional status of individuals with diabetes and prediabetes, which is computed using albumin, weight, and height. The GNRI (14) was defined as GNRI = [1.489 × serum albumin (g/L)] + [41.7 × weight (kg)/ideal weight (kg)], ideal weight = 22 × height (m) × height (m). If a participant’s weight was higher than the ideal weight, the weight/ideal weight ratio was regarded as 1. Although there is no universally accepted criterion for categorizing GNRI, we classified it into two groups based on previous literature (18, 24), using a cutoff value of 98: low GNRI group (<98) and high GNRI group (≥98).

### 2.3 Determination of mortality

All participants in our study were eligible for mortality follow-up. The primary outcomes were all-cause and cardiovascular mortality. The National Center for Health Statistics (NCHS) linked data with death certificate records by matching identification codes from the National Death Index (NDI). Consequently, we obtained mortality details from https://www.cdc.gov/nchs/data-linkage/mortality.htm. We used the *MORTSTAT* variable to ascertain each participant’s final survival status, which is assigned a vital status code (0 = assumed alive, 1 = assumed deceased), and the *UCOD_LEADING* variable to identify the leading cause of death code. All-cause mortality was defined as any reason for death. Cardiovascular mortality was defined according to the 10th revision of the International Classification of Diseases (ICD-10) coding, including I00–I09, I11, I13, and I20–I51. The follow-up time (*PERMTH_INT*) was calculated from each participant’s initial interview date to the end of the mortality period or December 31, 2018.

### 2.4 Covariates

This study gathered data on various factors from the NHANES Mobile Examination Center questionnaire and examination measurements, including gender, age, education level (less than high school, high school or equivalent, college or above) (25), race (Mexican American, other Hispanics, non-Hispanic White, non-Hispanic Black, other races), family income-to-poverty ratio (PIR), body mass index (BMI), HbA1c, marital status (living with a partner, lonely) (25), drinking status, and smoking status. PIR was categorized as low (<1.3), middle (1.3–3.5), and high (≥3.5) (26). BMI was divided into normal (<25 kg/m^2^), overweight (25–30 kg/m^2^), and obese (≥30 kg/m^2^) (27). Drinking status was categorized as never, former, or current drinking. Smoking status was categorized as never smoked, former smoker, and current smoker (18, 28).

### 2.5 Statistical analysis

In the analysis, we weighted the sample to ensure it accurately represented the whole US population. The baseline characteristics were presented and divided into two groups by GNRI. Continuous variables were described as mean ± standard deviation, while categorical variables were shown as numbers and percentages. To compare differences between the GNRI groups, the chi-squared test or Wilcoxon rank-sum test was applied for categorical and continuous variables, respectively.

We performed weighted multivariate Cox regression analyses to estimate hazard ratios (HRs) and 95% confidence intervals (CIs) for the relationship between GNRI and all-cause and cardiovascular mortality. The study was conducted across three progressively adjusted models: Model 1 (unadjusted), Model 2 (adjusted for gender, age, and race), and Model 3 (further adjusted for education level, PIR, BMI, HbA1c, marital status, drinking status, and smoking status).

Furthermore, we employed Kaplan–Meier survival analyses to evaluate the survival probabilities concerning all-cause and cardiovascular death. To deeper explore the dose–response relationships between GNRI and mortality outcomes in individuals with prediabetes and diabetes, we utilized a restricted cubic spline (RCS) model with four knots. Additionally, subgroup analyses and interaction tests were conducted on the fully adjusted model to investigate the heterogeneity of these associations across different subgroups.

All statistical analyses were performed using R version 4.2.2, and *p* < 0.05 was considered statistically significant.

## 3 Results

### 3.1 Baseline characteristics

A total of 7,640 participants from NHANES 2005–2018 were included in our study, with 5,184 diagnosed with prediabetes and 2,456 with diabetes. Table 1 displays the characteristics of the entire cohort stratified by their GNRI classification. Specifically, 6,988 participants were categorized in the high GNRI group, while 652 were in the low GNRI group. The majority of the cohort were male and aged between 45 and 64 years. Participants in the low GNRI group were more prone to be female, 45–64 years old, non-Hispanic White, obese, living with a partner, current drinkers, never smoked, and have college or above education, a low PIR, and higher HbA1c (*p* < 0.05). The low GNRI groups exhibited higher risks of all-cause and cardiovascular mortality compared to the high GNRI group.

**Table 1:** Baseline characteristics of participants.

Characteristic	Overall (*n* = 7,640)	High GNRI (*n* = 6,988)	Low GNRI (*n* = 652)	*p-*value
Gender				**<0.001**
Female	3,464 (45.3%)	3,034 (43.4%)	430 (66.0%)	
Male	4,176 (54.7%)	3,954 (56.6%)	222 (34.0%)	
Age				**0.026**
<45 years	2,171 (28.4%)	2,012 (28.8%)	159 (24.4%)	
45–64 years	2,988 (39.1%)	2,739 (39.2%)	249 (38.2%)	
≥65 years	2,481 (32.5%)	2,237 (32.0%)	244 (37.4%)	
Education level				**<0.001**
Less than high school	2,111 (27.6%)	1,884 (27.0%)	227 (34.8%)	
High school or equivalent	1,843 (24.1%)	1,687 (24.1%)	156 (23.9%)	
College or above	3,686 (48.2%)	3,417 (48.9%)	269 (41.3%)	
Race				**<0.001**
Mexican American	1,253 (16.4%)	1,174 (16.8%)	79 (12.1%)	
Other Hispanic	707 (9.3%)	657 (9.4%)	50 (7.7%)	
Non-Hispanic White	3,489 (45.7%)	3,204 (45.9%)	285 (43.7%)	
Non-Hispanic Black	1,523 (19.9%)	1,317 (18.8%)	206 (31.6%)	
Other races	668 (8.7%)	636 (9.1%)	32 (4.9%)	
PIR				**<0.001**
Low	2,424 (31.7%)	2,166 (31.0%)	258 (39.6%)	
Middle	2,956 (38.7%)	2,726 (39.0%)	230 (35.3%)	
High	2,260 (29.6%)	2,096 (30.0%)	164 (25.2%)	
BMI				**<0.001**
Normal	1,660 (21.7%)	1,502 (21.5%)	158 (24.2%)	
Overweight	2,613 (34.2%)	2,484 (35.5%)	129 (19.8%)	
Obese	3,367 (44.1%)	3,002 (43.0%)	365 (56.0%)	
HbA1c	5.70 ± 1.10	5.70 ± 1.05	5.80 ± 1.59	**<0.001**
Marital status				**<0.001**
Lonely	2,750 (36.0%)	2,465 (35.3%)	285 (43.7%)	
Living with a partner	4,890 (64.0%)	4,523 (64.7%)	367 (56.3%)	
Drinking status				**<0.001**
Never drinking	1,123 (14.7%)	1,017 (14.6%)	106 (16.3%)	
Former drinking	1,168 (15.3%)	1,026 (14.7%)	142 (21.8%)	
Current drinking	5,349 (70.0%)	4,945 (70.8%)	404 (62.0%)	
Smoking status				**0.004**
Never smoked	3,911 (51.2%)	3,617 (51.8%)	294 (45.1%)	
Former smoker	2,219 (29.0%)	2,037 (29.1%)	182 (27.9%)	
Current smoker	1,510 (19.8%)	1,334 (19.1%)	176 (27.0%)	
All-cause mortality	1,210 (15.8%)	989 (14.2%)	221 (33.9%)	**<0.001**
Cardiovascular mortality	319 (4.2%)	259 (3.7%)	60 (9.2%)	**<0.001**
Diabetes	2,456 (32.1%)	2,170 (31.1%)	286 (43.9%)	**<0.001**
Prediabetes	5,184 (67.9%)	4,818 (68.9%)	366 (56.1%)	**<0.001**
GNRI	105.7 ± 4.9	105.7 ± 4.0	95.3 ± 3.3	**<0.001**

Means ± standard deviations for continuous; *n* (%) for categorical.

GNRI, Geriatric Nutritional Risk Index; PIR, income-to-poverty ratio; BMI, body mass index; HbA1c: glycated hemoglobin; CVD: cardiovascular disease.

Bold *P*-values indicate statistical significance (*P* < 0.05).

### 3.2 Association of GNRI with mortality outcomes

**Figure 2:**
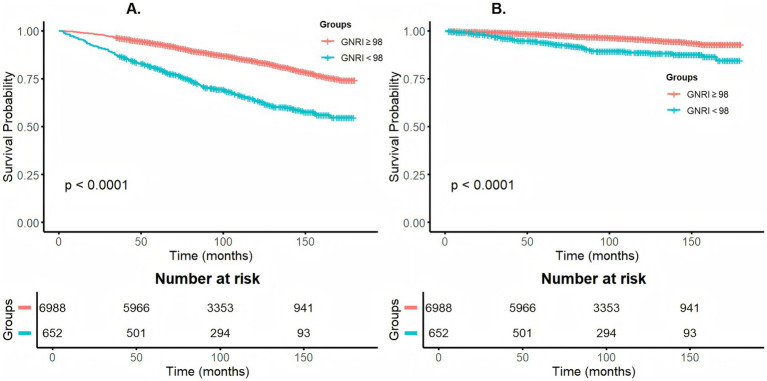
Kaplan–Meier analyses for all-cause **(A)** and cardiovascular mortality **(B)** among participants with prediabetes and diabetes.

Over a median follow-up period of 8.00 years (IQR: 5.17–11.08), 1,210 all-cause mortality events were recorded, including 319 deaths due to cardiovascular causes. Cox proportional hazards regression revealed a significant inverse association between GNRI and the risk of both all-cause and cardiovascular death, whether GNRI was treated as a continuous or categorical variable. In the fully adjusted model, each unit increase in GNRI corresponded to an 8% reduction in all-cause mortality risk and a 9% reduction in cardiovascular mortality risk. Stratified analyses revealed that individuals in low GNRI exhibited a 2.50-fold (95% CI: 2.14–2.92, *p* < 0.001) and 2.78-fold (95% CI: 2.04–3.77, *p* < 0.001) increased risk of all-cause and cardiovascular mortality, respectively, compared to those with high GNRI (Table 2). Furthermore, Kaplan–Meier survival curves confirmed that the survival probability was significantly greater in the high GNRI group than in the low GNRI group for both outcomes (*p* < 0.001) (Figure 2).

**Table 2:** Association of GNRI with all-cause and cardiovascular mortality.

Variable	Model 1		Model 2		Model 3	
	HR (95% CI)	*p*-value	HR (95% CI)	*p*-value	HR (95% CI)	*p*-value
All-cause mortality						
GNRI	0.91 (0.90, 0.92)	<0.001	0.91 (0.90, 0.93)	<0.001	0.92 (0.91, 0.94)	<0.001
GNRI group						
High GNRI	Ref.		Ref.		Ref.	
Low GNRI	2.97 (2.55, 3.46)	<0.001	2.96 (2.57, 3.42)	<0.001	2.50 (2.14, 2.92)	<0.001
Cardiovascular mortality						
GNRI	0.90 (0.88, 0.92)	<0.001	0.89 (0.87, 0.92)	<0.001	0.91 (0.88, 0.94)	<0.001
GNRI group						
High GNRI	Ref.		Ref.		Ref.	
Low GNRI	3.26 (2.48, 4.29)	<0.001	3.28 (2.44, 4.39)	<0.001	2.78 (2.04, 3.77)	<0.001

Model 1: not adjusted.

Model 2: adjusted for gender, age, and race.

Model 3: adjusted for all variables including gender, age, race, education level, PIR, BMI, HbA1c, marital status, drinking status, and smoking status.

GNRI, Geriatric Nutritional Risk Index; HR, hazard ratio; CI, confidence interval; PIR, income-to-poverty ratio; BMI, body mass index; HbA1c, glycated hemoglobin.

### 3.3 Dose–response relationship analysis

**Figure 3:**
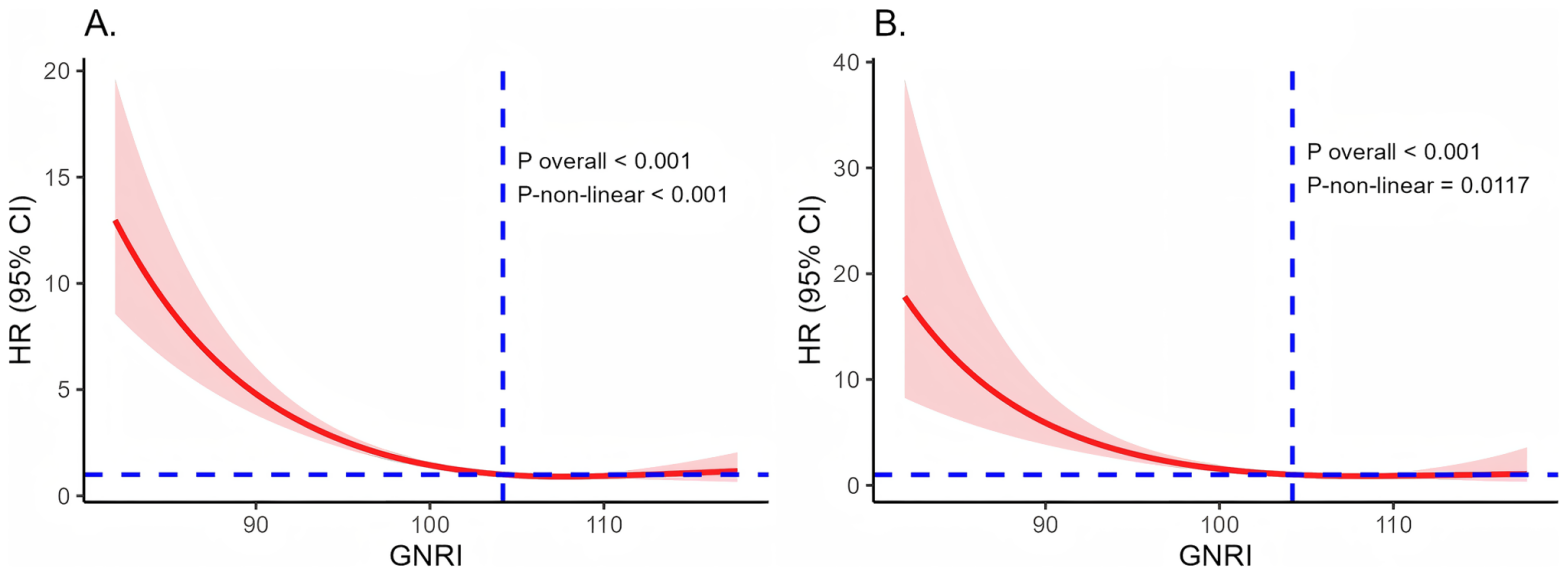
Dose–response relationships of GNRI and all-cause **(A)** and cardiovascular mortality **(B)**. Adjusted for gender, age, race, education level, PIR, BMI, marital status, drinking status, and smoking status. HR, hazard ratio; CI, confidence interval; GNRI, Geriatric Nutritional Risk Index; PIR, income-to-poverty ratio; BMI, body mass index.

To investigate the dose–response relationships between the GNRI and mortality rates among individuals with prediabetes and diabetes, we employed RCS analysis within a fully adjusted Cox regression model. The RCS curves revealed nonlinear relationships between GNRI and both all-cause and cardiovascular mortality (A: *p*-non-linear <0.001, *p* overall <0.001; B: *p*-non-linear = 0.0117, *p* overall <0.001). A significant trend was observed, with all-cause and cardiovascular mortality risk decreasing substantially as GNRI increased, particularly for GNRI values below 104.19 (Figure 3).

### 3.4 Subgroup analysis

**Figure 4:**
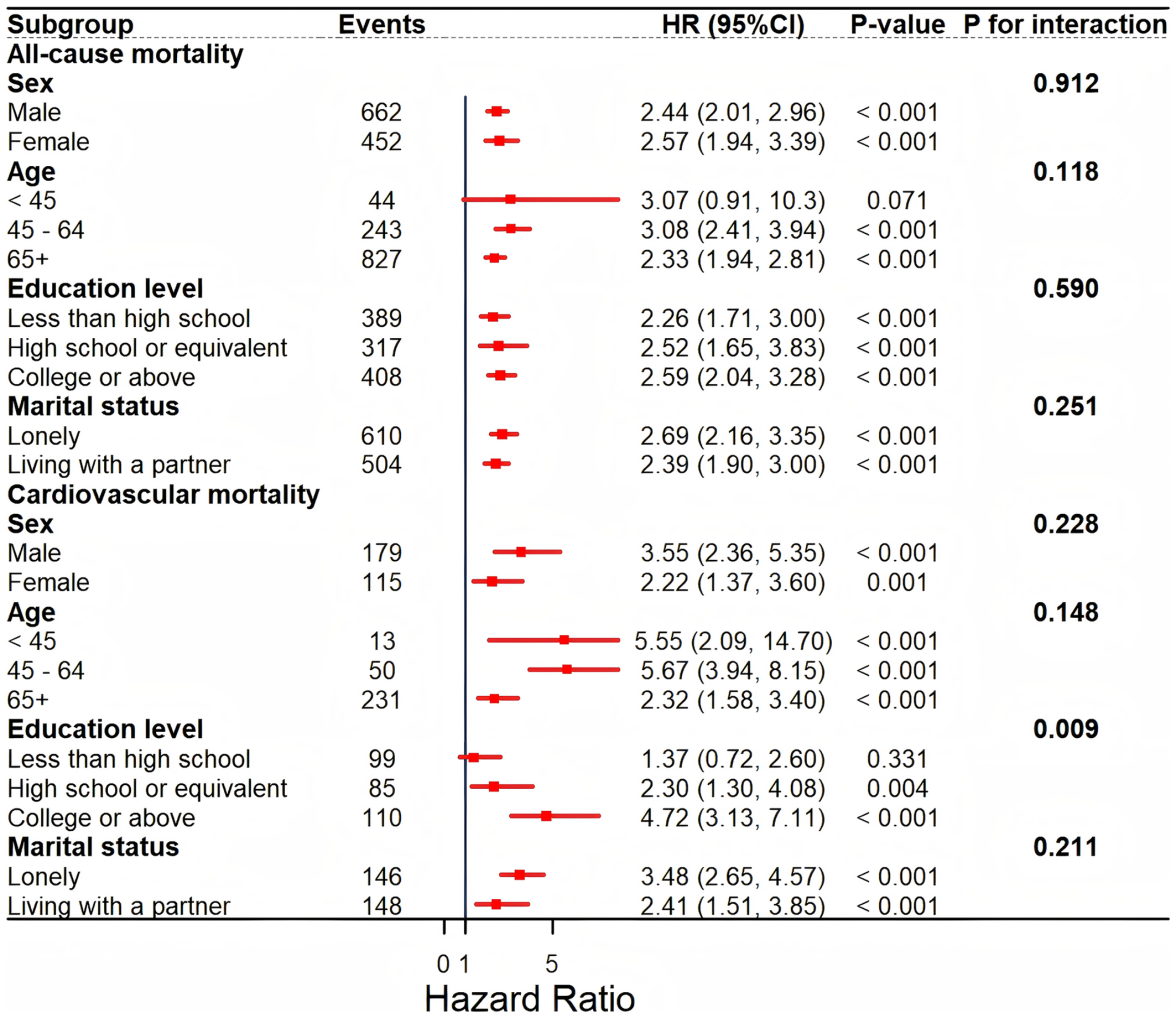
Subgroup analysis of the associations between GNRI and all-cause and cardiovascular mortality.

We conducted a stratified analysis further to investigate the relationship between GNRI and mortality outcomes, stratifying participants by gender, age, education level, and marital status (Figure 4). Interaction tests indicated no significant differences in the relationship between GNRI and all-cause mortality across these subgroups, suggesting a stable relationship in the populations with prediabetes and diabetes (all *p* for interaction >0.05). For cardiovascular mortality, interaction terms for age, gender, and marital status were not statistically significant (*p* for interaction >0.05), indicating stability across these subgroups. However, education level showed a significant interaction with GNRI (*p* for interaction = 0.009). The relationship of GNRI with cardiovascular mortality was significant in individuals with prediabetes and diabetes with a high school education or above, while no significant association was found in those with less than a high school education. This finding highlights the potential influence of education level on the relationship between GNRI and cardiovascular mortality. Moreover, we comprehensively examined the associations under different glycemic metabolic states and BMI. As shown in Supplementary Table S2, GNRI was significantly associated with both all-cause and cardiovascular mortality in both the prediabetes and diabetes groups, and no significant interaction was observed between glycemic metabolic states and the association of GNRI with mortality outcomes (*p* for interaction <0.05). It also showed that GNRI remains a reliable indicator of nutritional risk even in the presence of obesity (Supplementary Table S5). This additional analysis supports the validity of our approach and reinforces the applicability of the GNRI across various glycemic metabolic and body weight statuses.

## 4 Discussion

Our study advances this field by including individuals with prediabetes, thereby addressing the entire spectrum of diabetes and its early stages. Utilizing a nationally representative NHANES cohort with extended follow-up, we demonstrated that GNRI levels exhibited a negative association with the risks of all-cause and cardiovascular mortality among individuals with diabetes and prediabetes in the United States. This association was consistent across various subgroups. Additionally, dose–response analysis identified nonlinear associations between GNRI and mortality outcomes. Interaction tests identified education level as a significant interaction in the relationship between GNRI and cardiovascular mortality. These findings underscore the importance of early nutritional assessment and intervention in diabetes and prediabetes management, providing actionable insights for clinical practice and public health strategies.

Due to the effects of diet on inflammatory response, abnormal glucose metabolism, insulin resistance, and so on (29, 30), dietary management serves as one of the five cornerstones of lifestyle therapy for patients with prediabetes and diabetes, yet malnutrition can accelerate disease progression and increase mortality risk. Previous studies have established a clear link between poor nutritional status and increased mortality risk (31). For instance, Bonilla-Palomas et al. (32) and Arques et al. (33) utilized serum albumin level as a biomarker for nutritional status, demonstrating that hypoalbuminemia in acute heart failure patients was associated with higher hospital mortality rates. Similarly, large cohort studies and meta-analyses have identified hypoalbuminemia as a robust predictor of increased all-cause and cardiovascular mortality in hospitalized and non-hospitalized patients, regardless of comorbidities (34). In addition to serum albumin, BMI was commonly employed to evaluate nutritional status. Elevated BMI is linked to a heightened risk of several chronic diseases (3), such as cardiovascular disease, type 2 diabetes, chronic kidney disease, cancers, and musculoskeletal disorders. These conditions collectively contribute to increased global mortality rates (35). However, these traditional indicators have limitations in comprehensively assessing nutritional risk, underscoring the need for more integrated and multidimensional approaches.

The GNRI, which combines albumin levels with weight and height, offers a more reliable assessment of nutritional status, minimizing the effects of confounding factors on serum albumin or BMI alone. GNRI’s comprehensive nature effectively captures both acute stimuli and chronic reactions (36, 37). Recent studies suggest that it may also be applicable to younger populations, providing a comprehensive nutritional assessment tool for a broader demographic (38). Derived from but different from the Nutritional Risk Index (NRI) (39), GNRI is calculated using ideal body weight (40) rather than usual body weight. The Mini Nutritional Assessment (MNA) and the NRS-2002 are commonly employed to evaluate nutritional status. However, the MNA, despite its validated in a variety of settings, has a slightly weaker prognostic power due to its 30-question format (41). The NRS-2002 has a subjective nature and an inevitable requirement for reporting eating habits (42). Overall, the GNRI offers a highly convenient and accurate assessment of the nutritional status across a broad demographic.

Our findings indicated a negative relationship between GNRI and the rate of all-cause and cardiovascular mortality among individuals with diabetes and prediabetes in the United States. This relationship remained significant even after stratified analyses by gender, age, education level, and marital status. In the fully adjusted model, individuals in the low level of GNRI had a 1.50-fold increased risk of all-cause mortality and a 1.78-fold increased risk of cardiovascular mortality compared to those in the high GNRI group. Similarly, the Fukushima cohort study included 946 type 2 diabetes patients and defined renal, cardiovascular events, and all-cause death as endpoints. Their finding demonstrated that poor nutritional status, assessed by GNRI, has an association with adverse outcomes among diabetic individuals (43). Another analysis focusing on osteosarcopenia in older diabetic patients also showed that GNRI has a comprehensive clinical evaluation role of nutritional status and is helpful for early identification of those at high risk for osteosarcopenia (44). Additionally, studies concerning chronic kidney disease patients in the United States and the United Kingdom have shown that GNRI has a strong predictive capacity for the incidence of CKD and the risk of mortality (24). Our results are consistent with these previous studies.

Despite there may be differences in disease progression, development, and complications between prediabetes and diabetes, the predictive trends of the GNRI with respect to mortality risk remains similar across these two groups. This consistency is likely due to the shared underlying pathophysiological mechanisms that characterize both conditions. Strict restrictions on carbohydrate intake, coupled with an increased demand for dietary protein, have led to a growing prevalence of malnutrition in patients with diabetes and prediabetes. The relationship between abnormal glucose regulation and malnutrition is complex and multifaceted, with numerous studies shedding light on the underlying mechanisms that exacerbate each other. Consistent with the findings reported by Bourke et al. (45), immune dysfunction is both a cause and consequence of malnutrition. Malnutrition is associated with inflammatory and immune responses, which leads to increased levels of systemic proinflammatory mediator and immune cell activation (46). Similarly, Wang et al. (47) has the same finding that diabetic patients with elevated high high-sensitivity C-reactive protein level and malnutrition had a significantly higher risk of all-cause mortality compared to those with only one of these conditions. Inadequate dietary intake of essential nutrients can worsen insulin dysregulation and contribute to the progression of diabetes-related complications and MetS. Furthermore, malnutrition, particularly protein and calorie deficiencies, reduces the availability of essential amino acids and glucose, both of which are critical signals for insulin release. Chronic malnutrition, especially in the context of diabetes or prediabetes, can further impair β-cell and pancreatic activity (46). Additionally, chronic malnutrition can lead to hypoalbuminemia, which may result in oedema, especially in the gastrointestinal tract. The breakdown of the gastrointestinal barrier, exacerbated by malnutrition, increases the translocation of bacteria and toxins, further intensifying inflammatory reactions (48). Ultimately, it accelerates the decline in life expectancy.

Our study, which involved weighting the data, was a large-scale population-based survey that provided nationally representative results, making the findings generalizable to the general US individuals with prediabetes and diabetes. We employed Cox regression, RCS regression, and stratified analyses to demonstrate that nutritional status serves as a modifiable factor in the development of mortality among these individuals. However, our study does have limitations. First, due to the limited sample size and follow-up period, the median survival time has not yet been reached, and the Kaplan–Meier survival curves for all-cause and cardiovascular mortality showed no difference. Future studies could incorporate a larger sample size and extended follow-up period to ensure that survival differences can be detected. Second, nutritional status was assessed using GNRI only at baseline. Changes in serum albumin and weight during the follow-up period may have affected the relationship between GNRI and mortality. Additionally, owing to the inherent limitations of the NHANES database, crucial metabolic indicators like diabetes duration, types of glucose-lowering medication, and comorbidities lack complete recorded data, making it impossible to adequately account for the influence of these factors.

## 5 Conclusion

The GNRI shows a tight negative connection with all-cause and cardiovascular mortality among individuals with prediabetes and diabetes. Improving the nutrition early may protect these populations’ survival rates. Incorporating the GNRI index into routine evaluation can help early identification and treatment of diabetes and prediabetes.

## Data Availability

Publicly available datasets were analyzed in this study. This data can be found at: National Health and Nutrition Examination Survey: https://wwwn.cdc.gov/nchs/nhanes/Default.aspx.
