# Supplementary material for: Geriatric Nutritional Risk Index and mortality in individuals with prediabetes and diabetes: a longitudinal cohort study
Source: Front Nutr. 2025 Jul 15;12:1625281. doi: 10.3389/fnut.2025.1625281 (PMC12303801; doi:10.3389/fnut.2025.1625281)
Supplement: Supplementary file 1 [file Table_1.DOCX]

# Supplementary

**Table S1** Baseline characteristics between prediabetics and diabetics.

**Table S2** Stratified analysis of the associations between GNRI and all-cause and cardiovascular mortality across different glycemic metabolic states.

**Table S3** Optimal GNRI cutoffs and AUC values across different subgroups.

**Table S4** Comparison of all-cause mortality associated with traditional GNRI cutoff (98) versus optimized cutoff (102.48).

**Table S5** Stratified analysis of the associations between GNRI and all-cause and cardiovascular mortality across BMI.

**Table S6** Comparison of nutritional indicators between participants with GNRI ≤ 104 and > 104.

**Table S7** Linear Regression of GNRI with Nutritional Indicators.

**Table S1** Baseline characteristics between prediabetics and diabetics.

| **Characteristic** | **Overall**  **(N = 7640)** | **Prediabetes**  **(N = 5184)** | **Diabetes**  **(N = 2456)** | ***P* Value** |
| --- | --- | --- | --- | --- |
| **Gender** |  |  |  | **0.005** |
| Female | 3464 (45.3%) | 2320 (44.8%) | 1 144 (46.6%) |  |
| Male | 4176 (54.7%) | 2864 (55.2%) | 1 312 (53.4%) |  |
| **Age** |  |  |  | **< 0.001** |
| < 45 years | 2 171 (28.4%) | 1 844 (35.6%) | 327 (13.3%) |  |
| 45-64 years | 2 988 (39.1%) | 1 978 (38.2%) | 1 010 (41.1%) |  |
| ≥ 65 years | 2 481 (32.5%) | 1 362 (26.3%) | 1 119 (45.6%) |  |
| **Race** |  |  |  | **0.022** |
| Mexican American | 1 253 (16.4%) | 826 (15.9%) | 427 (17.4%) |  |
| Other Hispanic | 707 (9.3%) | 463 (8.9%) | 244 (9.9%) |  |
| Non-Hispanic White | 3 489 (45.6%) | 2 439 (47.0%) | 1 050 (42.8%) |  |
| Non-Hispanic Black | 1 523 (19.9%) | 965 (18.6%) | 558 (22.7%) |  |
| Other Races | 668 (8.7%) | 491 (9.5%) | 177 (7.2%) |  |
| **Education level** |  |  |  | **< 0.001** |
| Less than high school | 2 111 (27.6%) | 1 297 (25.0%) | 814 (33.1%) |  |
| High school or equivalent | 1 843 (24.1%) | 1 226 (23.6%) | 617 (25.1%) |  |
| College or above | 3 686 (48.2%) | 2 661 (51.3%) | 1 025 (41.7%) |  |
| **PIR** |  |  |  | **< 0.001** |
| Low | 2 424 (31.7%) | 1 582 (30.5%) | 842 (34.3%) |  |
| Middle | 2 956 (38.7%) | 1 938 (37.4%) | 1 018 (41.4%) |  |
| High | 2 260 (29.6%) | 1 664 (32.1%) | 596 (24.3%) |  |
| **Weight** | 84.50 ± 22.68 | 83.00 ± 21.97 | 88.90 ± 24.03 | **< 0.001** |
| **BMI** | 29.26 ± 7.15 | 28.67 ± 6.89 | 31.40 ± 7.44 | **< 0.001** |
| **HbA1c** | 5.70 ± 1.10 | 5.50 ± 0.37 | 6.50 ± 1.66 | **<0.001** |
| **Marital status** |  |  |  | **0.041** |
| Lonely | 2 750 (36.0%) | 1 836 (35.4%) | 914 (37.2%) |  |
| Living with a partner | 4 890 (64.0%) | 3 348 (64.6%) | 1 542 (62.8%) |  |
| **Drinking status** |  |  |  | **< 0.001** |
| Never drinking | 1 123 (14.7%) | 683 (13.2%) | 440 (17.9%) |  |
| Former drinking | 1 168 (15.3%) | 742 (14.3%) | 426 (17.3%) |  |
| Current drinking | 5 349 (70.0%) | 3 759 (72.5%) | 1 590 (64.7%) |  |
| **Smoking status** |  |  |  | **< 0.001** |
| Never smoked | 3 911 (51.2%) | 2 688 (51.9%) | 1 223 49.8%) |  |
| Former smoker | 2 219 (29.0%) | 1 399 (27.0%) | 820 (33.4%) |  |
| Current smoker | 1 510 (19.8%) | 1 097 (21.2%) | 413 (16.8%) |  |
| **GNRI** | 105.73 ± 4.89 | 105.73 ± 4.74 | 104.24 ± 5.14 | **< 0.001** |
| **All-cause mortality** | 1 210 (15.8%) | 622 (12.0%) | 588 (23.9%) | **< 0.001** |
| **Cardiovascular mortality** | 319 (4.2%) | 156 (3.0%) | 163 (6.6%) | **< 0.001** |

Means ± standard deviations for continuous; n (%) for categorical.

Abbreviations: PIR, income-to-poverty ratio; BMI, body mass index; HbA1c, glycated hemoglobin; GNRI, geriatric nutritional risk index.

**Table S2** Stratified analysis of the associations between GNRI and all-cause and cardiovascular mortality across different glycemic metabolic states.

| **Subgroup** | **HR** | **95% CI** | ***P* value** | ***P* for interaction** |
| --- | --- | --- | --- | --- |
| **All-cause mortality** |  |  |  |  |
| Glycemic metabolic states |  |  |  | 0.147 |
| Prediabetics | 2.69 | 2.16, 3.36 | < 0.001 |  |
| Diabetics | 2.13 | 1.59, 2.85 | < 0.001 |  |
| **Cardiovascular mortality** |  |  |  |  |
| Glycemic metabolic states |  |  |  | 0.114 |
| Prediabetics | 3.44 | 2.54, 4.66 | < 0.001 |  |
| Diabetics | 2.10 | 1.36, 3.25 | < 0.001 |  |

Abbreviations: HR, Hazard Ratio; CI, Confidence Interval.

**Table S3** Optimal GNRI cutoffs and AUC values across different subgroups.

| **Subgroup** | **Threshold** | **AUC** |
| --- | --- | --- |
| **Age** |  |  |
| < 45 years | 102.75 | 0.635 |
| 45-64 years | 99.97 | 0.626 |
| ≥ 65 years | 101.65 | 0.595 |
| **Race** |  |  |
| Mexican American | 101.61 | 0.662 |
| Other Hispanic | 104.22 | 0.614 |
| Non-Hispanic White | 105.72 | 0.620 |
| Non-Hispanic Black | 99.37 | 0.586 |
| Other Races | 102.79 | 0.747 |
| \| **Glycemic metabolic states** \| \| --- \| |  |  |
| Prediabetes | 104.82 | 0.630 |
| Diabetes | 102.45 | 0.586 |

Abbreviations: AUC, area under curve.

**Table S4** Comparison of all-cause mortality associated with traditional GNRI cutoff (98) versus optimized cutoff (102.48).

|  | **HR** | **95% CI** | ***P* value** |
| --- | --- | --- | --- |
| Traditional cutoff | 2.97 | 2.50, 3.52 | < 0.001 |
| Optimized cutoff | 2.16 | 1.89, 2.46 | < 0.001 |

Abbreviations: HR, Hazard Ratio; CI, Confidence Interval.

**Table S5** Stratified analysis of the associations between GNRI and all-cause and cardiovascular mortality across BMI.

| **Subgroup** | **HR** | **95% CI** | ***P* value** | ***P* for interaction** |
| --- | --- | --- | --- | --- |
| **All-cause mortality** |  |  |  |  |
| BMI |  |  |  | 0.657 |
| Normal | 2.51 | 1.92, 3.27 | < 0.001 |  |
| Overweight | 2.75 | 2.15, 3.52 | < 0.001 |  |
| Obese | 2.37 | 1.78, 3.16 | < 0.001 |  |
| **Cardiovascular mortality** |  |  |  |  |
| BMI |  |  |  | 0.934 |
| Normal | 3.02 | 1.62, 5.64 | < 0.001 |  |
| Overweight | 2.96 | 1.90, 4.61 | < 0.001 |  |
| Obese | 2.74 | 1.73, 4.35 | < 0.001 |  |

Abbreviations: HR, Hazard Ratio; CI, Confidence Interval; BMI, body mass index.

**Table S6** Comparison of nutritional indicators between participants with GNRI ≤ 104 and > 104.

|  | **GNRI ≤ 104** | **GNRI > 104** | ***P* value** |
| --- | --- | --- | --- |
| Serum albumin | 39.28 ± 2.27 | 44.43 ± 2.21 | < 0.001 |
| BMI | 32.63 ± 8.72 | 29.19 ± 5.61 | < 0.001 |

Means ± standard deviations.

Abbreviations: GNRI, geriatric nutritional risk index; BMI, body mass index.

**Table S7** Linear Regression of GNRI with Nutritional Indicators.

|  | **β** | **95% CI** | ***P* value** |
| --- | --- | --- | --- |
| Serum albumin | 1.45 | 1.44, 1.47 | < 0.001 |
| BMI | -0.17 | -0.20, -0.15 | < 0.001 |

Abbreviations: GNRI, geriatric nutritional risk index; CI, Confidence Interval; BMI, body mass index.
